# Formation of Precipitation Ellipsoidal Disks and Spheres in the Wake of a Planar Diffusion Front

**DOI:** 10.1021/acs.jpclett.3c02295

**Published:** 2023-11-13

**Authors:** Szabolcs Farkas, Ferenc Gazdag, Márton Detrich, Márton Mészáros, Gábor Holló, Gábor Schuszter, István Lagzi

**Affiliations:** † Department of Physics, Budapest University of Technology and Economics, Műegyetem rkp. 3, H-1111 Budapest, Hungary; ‡ Mihály Fazekas High School, Horváth Mihály tér 8, H-1082 Budapest, Hungary; § Department of Fundamental Microbiology, University of Lausanne, CH-1015 Lausanne, Switzerland; ∥ Department of Physical Chemistry and Materials Science, University of Szeged, Rerrich Béla tér 1, H-6720 Szeged, Hungary; ⊥ ELKH-BME Condensed Matter Research Group, Budapest University of Technology and Economics, Műegyetem rkp. 3, H-1111 Budapest, Hungary

## Abstract

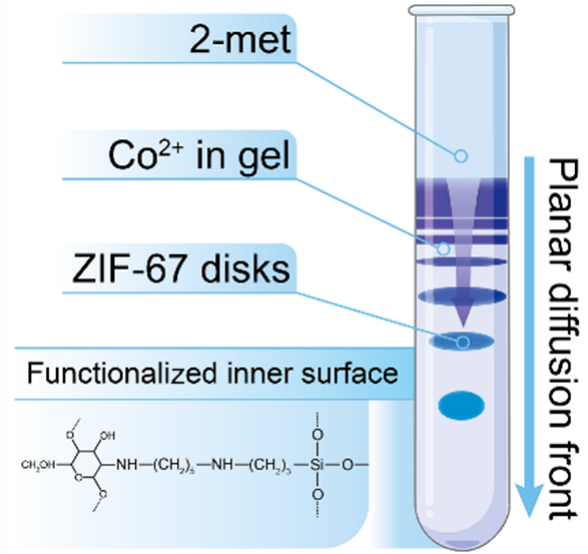

Pattern formation is one of the examples of self-organization. In the generation of patterns, the coupling between the mass transport of the chemical species and their chemical reactions plays an important role. Periodic precipitation (Liesegang phenomenon) is a type of pattern formation in which layered precipitation structures form in the wake of the diffusion front. Here, we show a new type of precipitation pattern formation in zeolitic imidazolate framework-67 in a solid hydrogel column in a test tube manifested in the generation of precipitation ellipsoidal disks and spheres in the wake of the planar diffusion front of the outer electrolyte (2-methylimidazole). To increase the probability of the emergence of ellipsoidal disks and spheres, the surfaces of the borosilicate test tubes were chemically treated and functionalized. To support the experimental findings, we developed a reaction–diffusion model that qualitatively describes the formation of precipitate ellipsoidal disks and spheres in a test tube.

Pattern formation is a frequent phenomenon in animate and inanimate systems. Usually, the pattern forms due to an interaction between the mass transport of chemical species and their reaction networks. One type of the most extensively investigated systems is the reaction–diffusion system, in which mass transport is realized by the diffusion of the chemical species. Turing patterns,^1−3^ Belousov–Zhabotinsky waves,^4^ and the Liesegang phenomenon^5,6^ are emblematic examples of pattern formation in reaction–diffusion systems. The Liesegang phenomenon (Liesegang banding or periodic precipitation) involves the formation of distinct precipitation bands or concentric rings in a gelled medium depending on the experimental setup.^6^ The most common experimental setup for the generation of Liesegang patterns is that one reagent of a precipitation reaction is homogeneously distributed in a solid hydrogel (e.g., agarose, gelatin) column (usually in a test tube) called the inner electrolyte, while the solution of another reagent is placed on top of the gel called the outer electrolyte. The concentration of the outer electrolyte is greater by at least 1 order of magnitude than that of the inner one, generating a planar diffusion and reaction front in the gel column. The pattern formation is generated by diffusion; therefore, the characteristic time to form macroscopic patterns (a few cm) is several days. Since the discovery of the Liesegang phenomenon, only patterns consisting of parallel precipitation bands or precipitation helicoids have been observed in test tubes in the wake of a planar diffusion front. The formation of parallel bands is a general process since the phenomenon is governed by the propagation of a planar diffusion front of the outer electrolyte. The formation of precipitation helicoids was understood by the permanent noise in the system that breaks the symmetry.^7^

Liesegang phenomena can occur in a wide range of chemical systems, including inorganic and coordination reactions as well as geochemical processes. Common examples include the precipitation of metal cations (such as Ag^+^, Pb^2+^, Fe^2+^, Ni^2+^, Ca^2+^) with inorganic anions (such as CrO_4_^2–^, Cr_2_O_7_^2–^, I^–^, OH^–^, CO_3_^2–^).^8−25^ Recently, a new class of the Liesegang pattern formation was discovered comprising the coordination reaction of metal ions with an organic linker forming the pattern of metal–organic frameworks (MOFs).^26−29,29^ MOFs are emerging materials in chemistry having unique properties due to their porous structure.^30−32^ These highly ordered, crystalline structures have been used in many applications such as gas storage and separation, catalysis, electronics, and targeted drug delivery.^33−38^ In recent studies, the formations of zeolitic imidazolate framework-8 and -67 (ZIF-8 and ZIF-67) were reported in a gel column.^27,28^ The pattern formation was manifested in the production of parallel precipitation bands of ZIFs.

In this work, we show that in the wake of a planar reaction front the formation of precipitation ellipsoidal disks and spheres can occur in the ZIF-67 system (composed of cobalt ion and 2-methylimidazole (2-met)) due to the interaction of the intermediate complex with the surface of the test tube. To investigate this effect, we chemically treated the inner surface of the glass to enhance the binding of the intermediate complex to the chemical groups on the surface of the glass. We observe that increasing the strength of the binding can favor the formation of spheres in the precipitate.

In a typical experiment, cobalt(II) sulfate (with concentrations ranging from 1 to 10 mM) was homogeneously distributed in a solid agarose gel (0.5% m/V prepared by using a 1:1 volumetric ratio of water and *N*,*N*-dimethylformamide (DMF)) placed in a cylindrical test tube (made of borosilicate glass with a diameter of 8.0 mm). After the gelation process, a solution of 2-met (1:1 water:DMF) was gently layered on the gel column. All experiments were carried out at room temperature (22 ± 0.5 °C) for 1 week. In the experiments, we used four types of test tubes: (i) native (untreated borosilicate) test tubes, (ii) test tubes treated with piranha solution, (iii) test tubes treated with piranha solution and then treated with a solution of concentrated sodium hydroxide, and (iv) test tubes treated with piranha solution followed by a solution of concentrated sodium hydroxide and then functionalized with chitosan. The details of the experimental procedure, treatment, and functionalization of the borosilicate glass can be found in the Supporting Information (SI) and Figure S1.

Figure 1 shows the typical periodic precipitation patterns formed in the native borosilicate test tubes. The macroscopic pattern consisted of a set of parallel precipitation bands, and the characteristic distance between two consecutive bands increased as the initial concentration of Co^2+^ was increased. This is the manifestation of the Matalon–Packter law, which states that the spacing coefficient of the pattern (calculated as a ratio of the distances of the two consecutive bands measured from the liquid–gel interface) depends on the initial concentration of the inner and outer electrolyte.^6,39,40^ First, we explored the effect of the anions of the cobalt salt on the pattern formation, and we used two additional salts, namely, cobalt acetate and nitrate. Figure S2 presents the generated patterns, showing that the anions dramatically affected the pattern structures. In the case of acetate, we could not produce any patterns at all, and using nitrate salt, the bands were generated only near the liquid–gel interface. This finding highlights the importance of the effect of background ions on the formation of ZIFs.^41^

**Figure 1:**
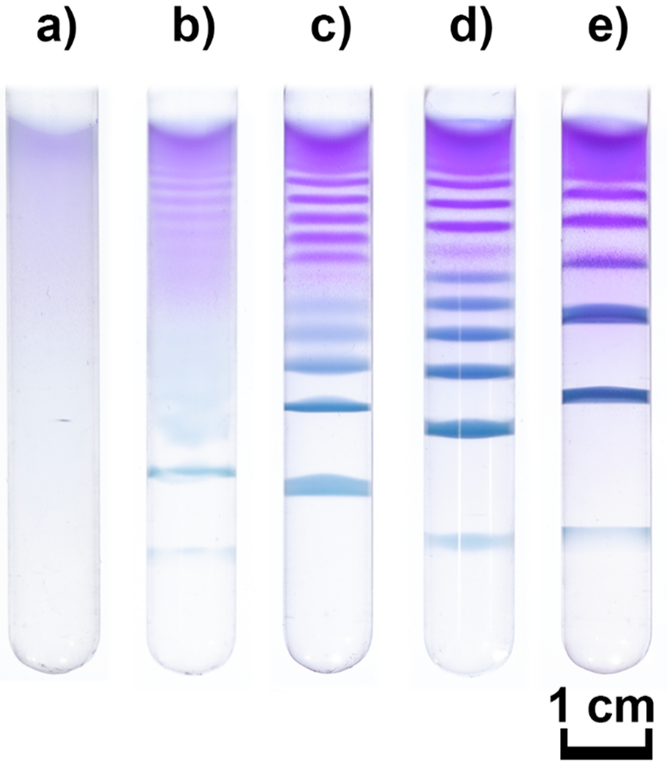
Periodic precipitation of ZIF-67 in the gel matrix in borosilicate test tubes after 1 week of reaction and diffusion of ZIF precursors at room temperature. The cobalt cations (cobalt sulfate) were homogeneously distributed in the agarose gel (0.5% m/V; DMF/H_2_O volumetric ratio was 1:1). (a) [Co^2+^]_0_ = 1.0 mM, (b) [Co^2+^]_0_ = 2.5 mM, (c) [Co^2+^]_0_ = 5.0 mM, (d) [Co^2+^]_0_ = 7.5 mM, and (e) [Co^2+^]_0_ = 10.0 mM. The concentration of the outer electrolyte (2-met) was 1.0 M.

In most inorganic systems, periodic precipitation can be achieved only if the metal cations diffuse in the gel containing anions. Usually, if the setup is reversed and anions diffuse into the gel matrix containing metal cations, then no pattern formation can be observed. This finding can be explained by the colloidal stability of the intermediate species that plays an important role in pattern formation. If cations diffuse into the gel from the high-concentration outer solutions, their local concentration in the gel is greater than that of the anions, and some cations can preferentially adsorb on the surface of colloidal particles, thus stabilizing the sols more than in the reverse case, when the concentration of the anions would be greater.^42^ Inspired by this fact, we carried out reversed experiments in which the 2-met was homogeneously distributed in the gel and cobalt cations diffused from outside. In this experiment, we observed in some cases only the formation of a few bands (Figure S3), and the pattern formation was not as pronounced as when 2-met was the outer electrolyte. This could be because the formation of ZIFs usually requires an excess of 2-met, which is especially hard to attain using a water-based solvent.^43^

We observed that in native borosilicate test tubes predominantly the banded structure appeared (Figure 2a); however, sometimes with a low probability (∼10%), the formation of precipitation ellipsoidal disks and spheres after the banded structure was observed (Figure S4). The surface of the borosilicate glass has a negative surface charge density, primarily through the dissociation of the terminal silanol groups. Therefore, we hypothesized that this effect could be due to the interaction of the negative surface of the test tube with the positively charged chemical species such as the cobalt ions and Co–2-met complex (intermediate). However, the role of the cobalt ions might be negligible since they were homogeneously distributed in the gel, and the pattern formation took place over 1 week. A hypothesized inhomogeneous distribution of cobalt ions, in conjunction with the planar diffusion front due to the binding of Co^2+^ to the surface, would be smoothened in ∼4.5 h (based on the calculation of the characteristic diffusion time of small, hydrated ions with a diffusion coefficient of 10^–9^ m^2^/s using 0.4 cm as the radius of the test tube). Most likely the important chemical species in the pattern formation, according to the symmetry breaking, is the other positively charged species, the Co–2-met complex, which forms by the coordination of the cation with 2-met. This complex has a positive charge ([Co(2-met)_4_]^2+^) because in water the p*K*_a_ of 2-met is ∼14, meaning that practically no 2-met molecules are deprotonated; therefore, they are electrically neutral.^44^ It is known that DMF (and other organic solvents) can weaken or strengthen the acidity or basicity of a chemical species due to the stabilization or destabilization of the charged or uncharged forms of the solute differently from water. However, we hypothesize that the presence of DMF decreases the p*K*_a_ of 2-met, but in the generated solvent environment, most of the 2-met molecules are still neutral.^45^ This complex is generated in the moving reaction front; therefore, there is not enough time to smoothen its concentration along the cross-section of the test tube as in the case of the cobalt ions. The positively charged intermediate can adsorb on the negatively charged glass surface, decreasing its concentration near the wall of the test tube. The precipitate formation starts where the concentration of the intermediate exceeds a certain threshold value, expected to be in the centerline of the tube, where the complex concentration is maximal, and it grows toward the wall. While the precipitation domain grows, it depletes the reagents and complex in its vicinity, resulting in the domain not reaching the wall of the tube, while forming rotationally symmetric objects like ellipsoidal disks and spheres. As the reaction front goes further along the tube, it reaches the threshold concentration later again in the centerline, and the process starts over.

**Figure 2:**
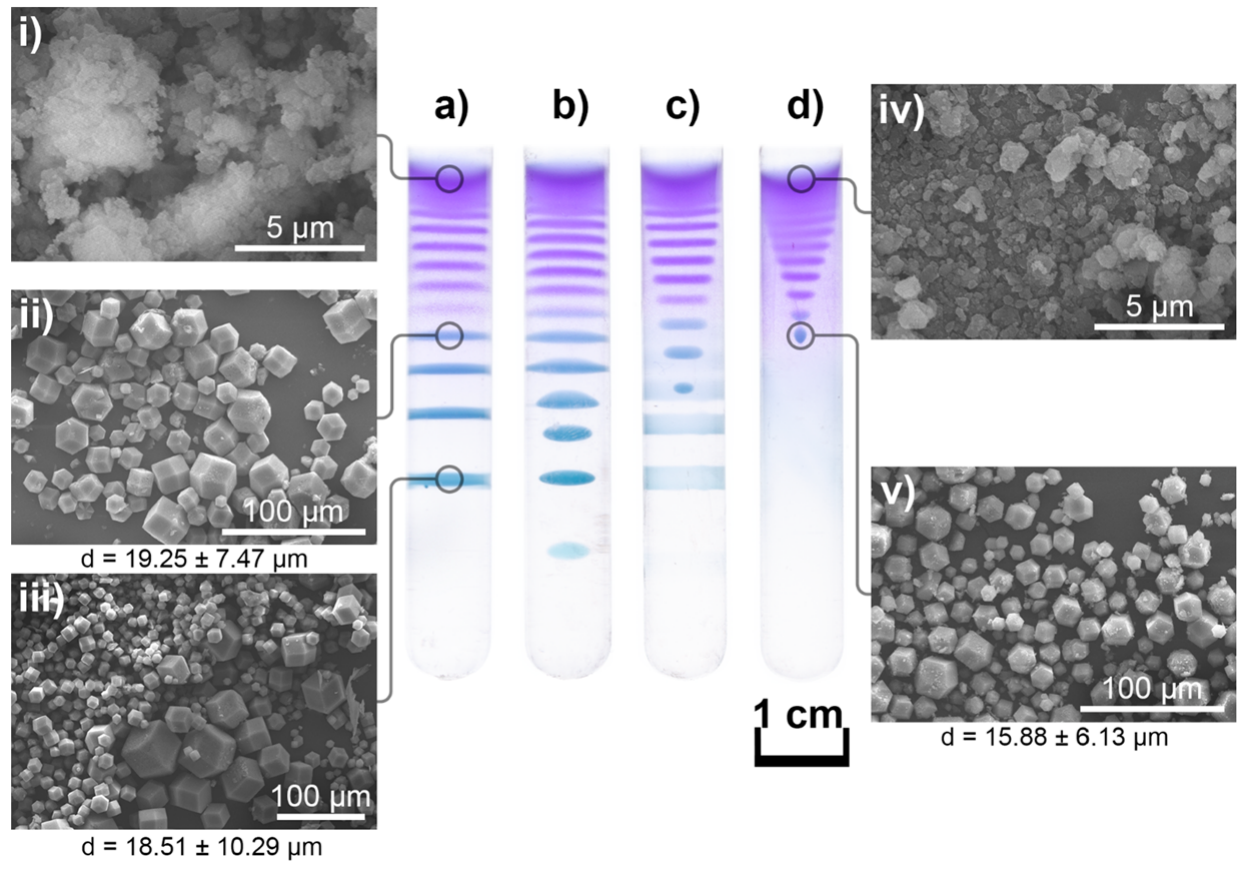
Periodic precipitation of ZIF-67 in the gel matrix after 1 week of the reaction and diffusion of ZIF precursors at room temperature: (a) the native borosilicate test tube, (b) the borosilicate test tube treated with a piranha solution, (c) the borosilicate test tube treated with a piranha solution followed by the treatment using a sodium hydroxide solution, and (d) the borosilicate test tube treated with piranha and sodium hydroxide solutions followed by the functionalization with chitosan. The cobalt cations ([Co^2+^]_0_ = 5 mM) were homogeneously distributed in the agarose gel (0.5% m/V, DMF/H_2_O volumetric ratio was 1:1). The concentration of the outer electrolyte (2-met) was 1.0 M. Panels i–v show the SEM micrographs of the ZIF-67 crystals extracted from the domains of the agarose gel indicated in (a) and (d).

To verify our assumption, we carried out experiments in three types of modified glass test tubes. In the first two sets, we increased the number of negatively charged terminal groups with chemical treatments. Ultimately, in the third setup, we functionalized the glass surface by chitosan having the ability to bind strongly to cobalt cations.^46^ First, the test tubes were treated with piranha solution, which is highly oxidative by removing all organic residues from the surface and hydroxylates the surface by increasing silanol groups and Si–O species on the glass surface.^47^ In these experiments, we obtained parallel bands followed by ellipsoidal disk-like zones which did not attach to the wall of the tubes (Figure 2b) with a probability of ∼50%. In a second set of experiments, after treatment using piranha solution, the surface of the test tubes was also treated with a sodium hydroxide solution. This caused the head groups of the borosilicate surface to become more negative compared to the previous cases due to the hydrolysis of the silicon oxide at the surface creating silanol and silanol salt groups.^48^ In this case, the detachment of the precipitation ellipsoidal disks started closer to the liquid–gel interface (Figure 2c), and the probability of the formation of disks and spheres was again at 50%. However, once the glass surface was functionalized with chitosan, a set of ellipsoidal disks and spheres appeared in the test tube (Figure 2d) with a very high probability (∼90%).

To get insight into the microscopic structure of the formed ZIF-8 crystals to use them in various applications (e.g., catalyst, gas storage, and drug delivery),^49^ we performed scanning electron microscopy (SEM) measurements (see the SI for the sample preparation and description of the measurement). Panels (i–v) in Figure 2 show the SEM micrographs of the formed particles in a native borosilicate test tube and a test tube whose inner surface was functionalized with chitosan. In both cases, the crystals generated near the liquid–gel interface were small and amorphous (Figure 2, Panels (i) and (iv)). However, farther from the interface, well-shaped crystals were formed. We compared the average size and dispersity of the particles (characterized by the standard deviation of the samples) in both cases at the same distance measured from the liquid–gel interface where the precipitation spheres were produced (∼2 cm). We found that the parallel precipitation bands consist of crystals with a size of 19.2 ± 7.5 μm (Figure 2, Panel (ii)); while the precipitation spheres contain particles having slightly lower dispersity with a size of 15.9 ± 6.1 μm (Figure 2, Panel (v)). In the case of a native borosilicate tube, the dispersity of the ZIF-67 crystals increased in the distance measured from the liquid–gel interface (Figure 2, Panels (i–iii)). This finding is in good accordance with the results obtained in other systems (formation of inorganic particles and gold nanoparticles).^50,51^

To support the fact that the surface of the test tube plays an important role in the formation of elliptical disks and spheres, control experiments were carried out in plastic tubes (Falcon = 15 mL). In these cases, the parallel band pattern structure was always generated (Figure S5).

To illustrate our concept, we developed a reaction–diffusion model to qualitatively describe the experimental findings. The kinetic model of the formation of ZIF is based on the following chemical reactions and rates:
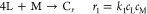
1
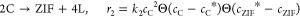
2
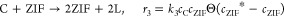
3where L, M, C, and ZIF and *c*_L_, *c*_M_, *c*_C_, and *c*_ZIF_ denote the linker, cobalt ion, Co–2-met complex, and the formed ZIF-67 and their concentrations, respectively. *k*_1_, *k*_2_, and *k*_3_ are the corresponding reaction rate coefficients. The first reaction (eq 1) describes the formation of the intermediate. The second and third reactions (eqs 2 and 3) represent the homogeneous and heterogeneous formation of ZIF-67, respectively. Θ is the Heaviside step function, and it reflects the fact that precipitation occurs only if the concentrations of the intermediate species and ZIF-67 reach threshold concentrations (*c*_C_*and *c*_ZIF_*). These threshold-limited steps are usual in mathematical models of precipitation.^6^ In the model, we assume that the intermediate species reversibly adsorb on the inner wall of the test tube using the following reaction steps:
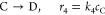
4
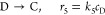
5Here, D and *c*_D_ are the adsorbed intermediate species and its concentration, respectively. The details of the numerical simulations can be found in the SI.

Figure 3 shows the results of numerical simulations. One can see that the kinetic model extended with the reversible adsorption of the intermediate species could qualitatively describe the experimental results. At the early stage of the process, the formed precipitate filled the whole test tube in width. Later, however, the preferential adsorption of the formed intermediate on the wall of the test tube distorted the homogeneous distribution of the intermediate species generated in the wake of the planar diffusion front of the outer electrolyte. This involved the homogeneous precipitation (eq 2) occurring at the centerline of the test tube, and a precipitation sphere started to develop from the center.

**Figure 3:**
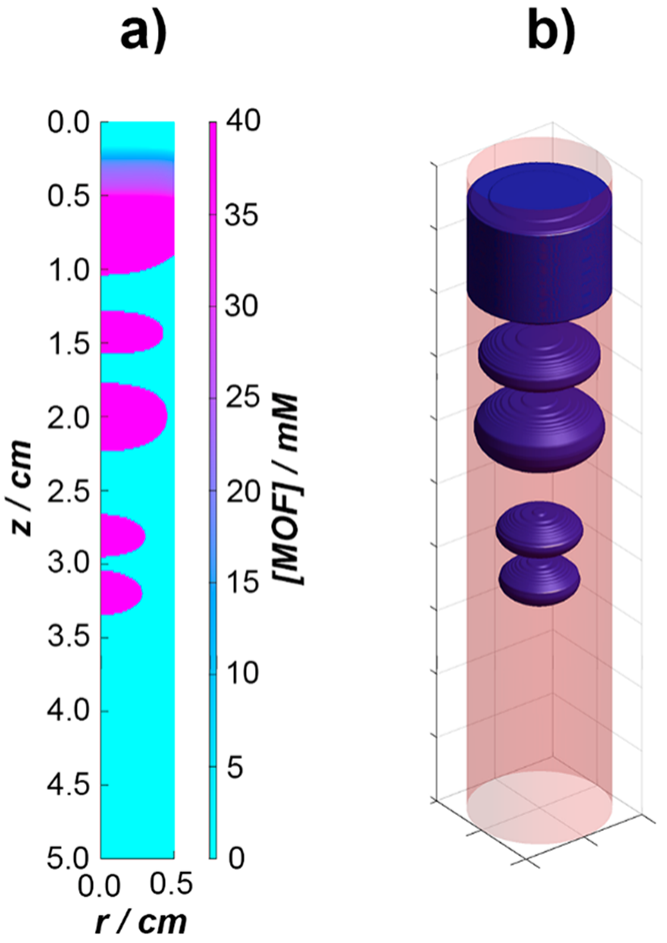
Result of a numerical simulation incorporating the reversible binding of the Co–2-met complex with the surface of the test tube; (a) the 2D pattern using a semipolar coordinate system and (b) a 3D representation of the generated pattern.

In this study, we presented an unexpected pattern formation in a reaction–diffusion system, namely, the formation of precipitation ellipsoidal disks and spheres in the wake of the planar diffusion front of the outer electrolyte (2-met). The generation of precipitation disks and spheres was due to symmetry breaking generated by the surface of the test tubes. We hypothesized that the formed intermediate species (having a positive charge) interacts with the surface of the wall of the test tube. To enhance the formation of ellipsoidal disks and spheres, we chemically treated and functionalized the inner surface of the borosilicate test tubes to increase the strength of the interaction of the cobalt intermediate complex with the terminal groups of the glass surface. The investigation showed that the particles generated in the precipitation spheres have lower polydispersity than those formed in parallel bands in native borosilicate test tubes. Based on the findings, we provided here a new strategy to design self-assembled rotationally symmetric precipitation structures in the wake of planar mass transport fronts.

## Supporting Information Available

Description of the experiments, treatment, functionalization of the inner surface of the test tubes, and sample preparation for SEM measurements; description of the reaction–diffusion model and Figures S1–S5 showing the steps of the functionalization of the glass surface, the effect of anions on the pattern formation, results of the revert experiments, the formation of the nonregular pattern in glass tubes, and the formation of the pattern in the Falcon tubes (PDF)
Transparent Peer Review report available (PDF)
