# Supplementary material for: Formation of Precipitation Ellipsoidal Disks and Spheres in the Wake of a Planar Diffusion Front
Source: J Phys Chem Lett. 2023 Nov 13;14(46):10382–7. doi: 10.1021/acs.jpclett.3c02295 (PMC10683069; doi:10.1021/acs.jpclett.3c02295)
Supplement: Supplementary file 1 — jz3c02295_si_001.pdf [file jz3c02295_si_001.pdf]

Supporting Information

Formation of Precipitation Ellipsoidal Disks and Spheres in the Wake of a Planar Diffusion Front

Szabolcs Farkas[1], Ferenc Gazdag[1,2], Márton Detrich[1,2], Márton Mészáros[1], Gábor Holló[3], Gábor Schuszter[4], István Lagzi[1,4]*

[1]Department of Physics, Budapest University of Technology and Economics, H-1111 Műegyetem rkp. 3, Budapest, Hungary

[2]Mihály Fazekas High School, H-1082, Horváth Mihály tér 8, Budapest, Hungary

[3]Department of Fundamental Microbiology, University of Lausanne, CH-1015, Lausanne, Switzerland

[4]Department of Physical Chemistry and Materials Science, University of Szeged, H-6720 Rerrich Béla tér 1, Szeged, Hungary

[5]ELKH-BME Condensed Matter Research Group, Budapest University of Technology and Economics, H-1111 Műegyetem rkp. 3, Budapest, Hungary

1. Experimental

1.1 Synthesis of ZIF-67 in agarose gel

A 0.5% m/V agarose solution was prepared by dissolving agarose powder in a 1:1 mixture of dimethylformamide (DMF) and water by heating it to 80 °C. After a clear solution was obtained an equal volume of cobalt sulfate solution was added while stirring. The solution was reheated to 80 °C and left to cool. After the solution reached around 60 °C, it was poured into test tubes so that 2/3rd of the tubes was filled with the gel. After the gels cooled down, they were kept at 4 °C to complete the gelation. After 24 hours the test tubes were warmed to room temperature and 2-methylimidazole (2-met) dissolved in a 1:1 mixture of dimethylformamide and water was poured on top of the gels.

1.2 Surface modification with piranha solution

To produce the piranha solution 30% m/m hydrogen peroxide solution was slowly added to concentrated sulfuric acid. The mixture was heated to 90 °C and was poured into test tubes. The solutions were kept at 100 °C in a heating block for 1 hour. After this procedure, test tubes were rinsed with water and filled with the prepared agarose solutions.

1.3 Surface modification with the solution of NaOH

Surface modification with the solution of NaOH was based on [Liu *et al.*] and carried out with test tubes modified Piranha-solution.[1] For this method 4 M NaOH solution was prepared and heated to 100 °C. The solution was then poured into test tubes and was kept at 100 °C in a heating block for 0.5 h. After the modification was complete the test tubes were rinsed with water and filled with the prepared agarose solutions.

1.4 Surface modification with chitosan

Test tubes modified with NaOH solution were used for this procedure (**1**). The following method is based on Liu *et al.* with certain modifications.[1] Test tubes were rinsed with water and dried at 70 °C. They were then filled with a 3:10 mixture of (3-aminopropyl)triethoxysilane (APES) and toluene and were left to react at 80 °C in a heating block for 24 hours (**2**). After the reaction, the test tubes were rinsed with toluene, dichloromethane, and acetone. The dried test tubes were filled with a 25% glutaraldehyde solution and were left to react for 1 hour at room temperature (**3**). When the reaction was complete the tubes were rinsed with methanol and were filled with a slightly acidic (4% V/V acetic acid (HAc)) solution of chitosan (2% m/V) and were left to react at room temperature for 1 hour (**4**). The test tubes were rinsed with water and filled with a 1% m/V solution of $NaBH_4$ and were left at room temperature for 1 hour (**5**). Finally, the test tubes were rinsed with water and filled with the prepared agarose gels.

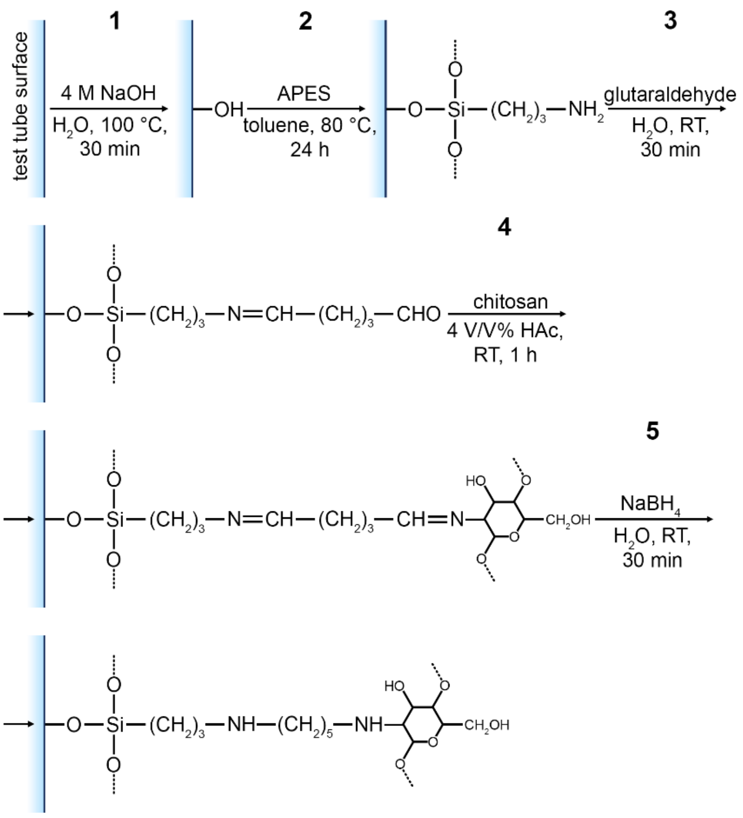

Figure S1 Steps of the surface modification with chitosan.

1.5 Samples preparation for scanning electron microscopy (SEM) measurement

The test tube containing the gel column was scratched with a glass cutting tool, and the tube was carefully broken. The gel was slid out and sliced up at the appropriate heights with a razor blade. The pieces of gel were put into Eppendorf tubes (2 mL) and dissolved in 1.5 mL DMF. The solution was centrifuged for 15 minutes at 10000 rpm (Hettich ROTOFIX 32A) and the supernatant was carefully removed. The remaining precipitate was suspended in 1.5 mL DMF and centrifuged again. These cleaning steps were repeated three times. After the final centrifugation step, the samples were dried at ambient conditions.

1.6 SEM measurement

The formed crystals were investigated via SEM (Hitachi S4700) by applying a secondary electron detector (Everhart–Thornley detector) and 20 kV accelerating voltage. The dry precipitate was placed onto conducting tape and gold sputtering was performed to maintain appropriate electrical conductance.

2. The numerical model

The reaction–diffusion model consists of the following second-order partial differential equations

$$\frac{\partial c_L}{\partial t} = D_L \nabla^2 c_L - 4r_1 + 4r_2 + 2r_3, \tag{S1}$$

$$\frac{\partial c_M}{\partial t} = D_M \nabla^2 c_M - r_1, \tag{S2}$$

$$\frac{\partial c_C}{\partial t} = D_c \nabla^2 c_C + r_1 - 2r_2 - r_3 - r_4 + r_5, \tag{S3}$$

$$\frac{\partial c_{ZIF}}{\partial t} = r_2 + r_3, \tag{S4}$$

$$\frac{\partial c_D}{\partial t} = r_4 - r_5 \tag{S5}$$

where c_L, c_M, c_C, c_{ZIF}, and c_D are the concentrations of the linker, cobalt ion, Co–2-met complex, the formed ZIF-67, and the concentration of the immobilized Co–2-met complex at the surface of the test tube. D_L, D_M, and D_c are the diffusion coefficients of the linker, cobalt ion, and Co–2-met complex, which were set to 10^{-9} m^2 s^{-1} in the simulations, and ∇ is the Nabla operator. The ZIF-67 and Co–2-met complex at the surface of the test tube could not diffuse. The threshold concentrations were $c_C{}^* = 5 \times 10^{-3}$ M and $c_{ZIF}{}^* = 4 \times 10^{-2}$ M. The meaning of the reaction rates (r_1, r_2, r_3, r_4, and r_5) is given in the main text, where the following reaction rate coefficients were used: $k_1 = 10^{-3}\ \mathrm{M^{-1}s^{-1}}$, $k_2 = 10^{-3}\ \mathrm{M^{-1}s^{-1}}$, $k_3 = 10^{-1}\ \mathrm{M^{-1}s^{-1}}$, $k_4 = 2 \times 10^{-3}\ \mathrm{s^{-1}}$, and $k_5 = 10^{-5}\ \mathrm{s^{-1}}$. We solved the set of partial differential equations (Equations S1-S5) numerically by using the method of lines technique using a semi-polar coordinate system. The finite difference spatial discretization method was combined with a forward Euler method for the integration in time of the set of ordinary differential equations.[2] We applied the following initial conditions $c_L(t = 0, x, r) = 0$, $c_M\ (t = 0, x, r) = 2 \times 10^{-2}$ M, $c_C\ (t = 0, x, r) = 0$, $c_{ZIF}(t = 0, x, r) = 0$, and $c_D(t = 0, x, r) = 0$ to reflect the initial experimental conditions, i.e., cobalt cations were uniformly distributed in the gel column. We used no-flux boundary conditions for all chemical species in the computational domain, except at the liquid–gel interface ($x = 0$, r). The applied boundary condition was the Dirichlet boundary condition ($c_L(t, x = 0, r) = 1$ M, $c_M(t, x = 0, r) = 0$, $c_C(t, x = 0, r) = 0$, $c_{ZIF}(t, x = 0, r) = 0$, and $c_D(t, x = 0, r) = 0$). The size of the rectangular computational domain was 0.5 × 5 cm with the grid spacing of $\Delta r = 1.25\times10^{-2}$ cm and $\Delta x = 6.25\times10^{-3}$ cm. The total time of the simulations was 3×10^5 s with a time step of 0.3 s.

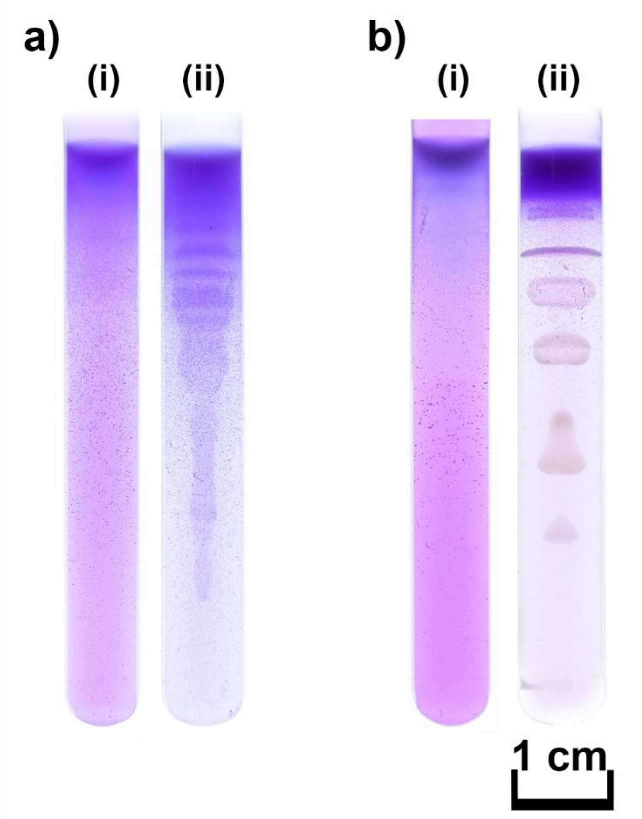

Figure S2 Periodic precipitation of ZIF-67 using various cobalt salts ((i) cobalt acetate, (ii) cobalt nitrate) in the gel matrix in borosilicate test tubes after 1 week of the reaction and diffusion of ZIF precursors at room temperature. The cobalt cations were homogeneously distributed in the agarose gel (0.5% m/V, DMF/H_2O volumetric ratio was 1:1). a) $[Co^{2+}]_0$ = 5.0 mM and b) $[Co^{2+}]_0$ = 10.0 mM. The concentration of the outer electrolyte (2-met) was 1.0 M.

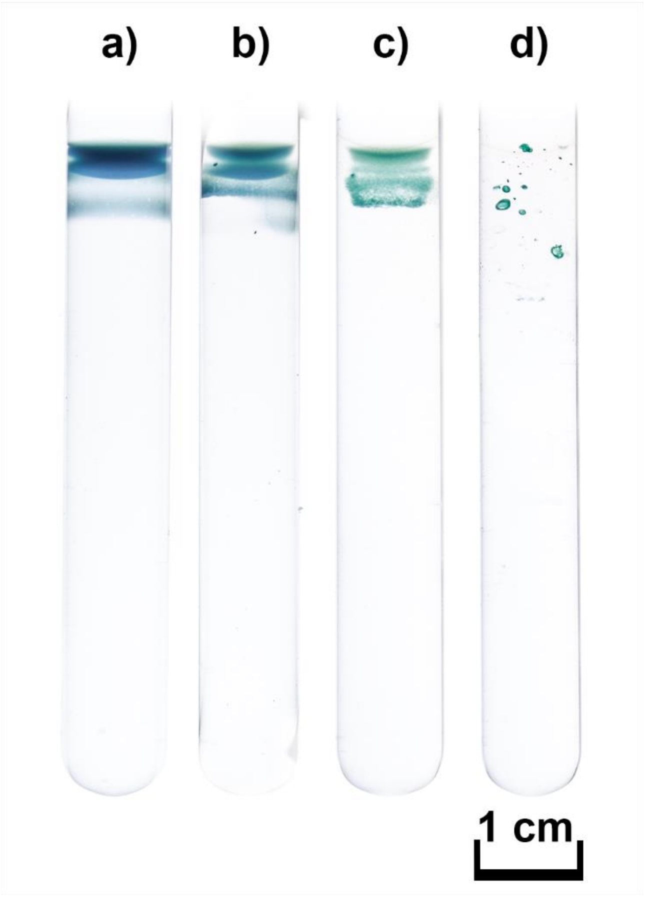

Figure S3 Periodic precipitation of ZIF-67 in the gel matrix in borosilicate test tubes after 1 week of the reaction and diffusion of ZIF precursors at room temperature. The 2-met was homogeneously distributed in the agarose gel (0.5% m/V, DMF/H_2O volumetric ratio was 1:1). a) $[\text{2-met}]_0$ = 50.0 mM, b) $[\text{2-met}]_0$ = 20.0 mM, c) $[\text{2-met}]_0$ = 10.0 mM and d) $[\text{2-met}]_0$ = 5.0 mM. The concentration of the outer electrolyte (cobalt sulfate) was 20 mM.

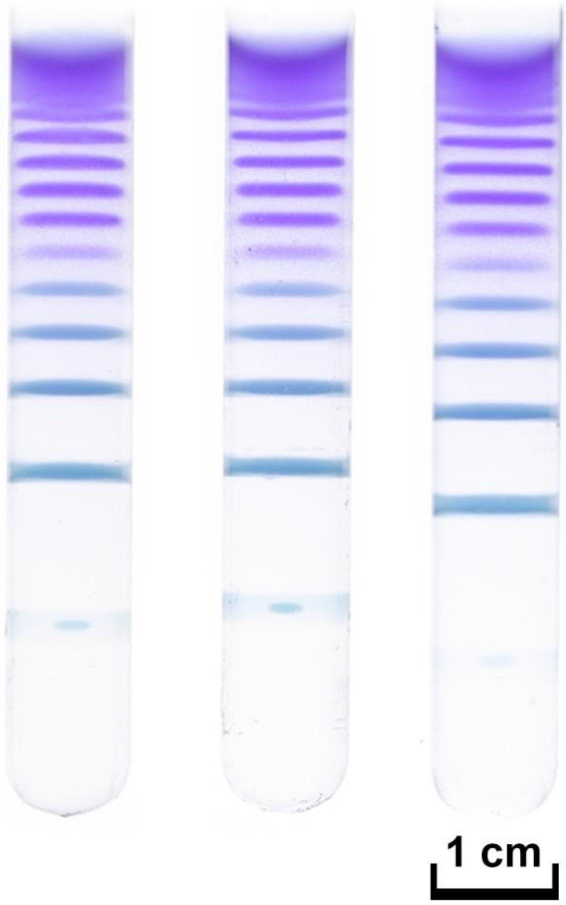

Figure S4 Formation of a non-regular pattern of ZIF-67 in the gel matrix in borosilicate test tubes after 1 week of the reaction and diffusion of ZIF precursors at room temperature. The cobalt cations ($[Co^{2+}]_0$ = 5 mM) were homogeneously distributed in the agarose gel (0.5% m/V, DMF/H_2O volumetric ratio was 1:1). The concentration of the outer electrolyte (2-met) was 1.0 M.

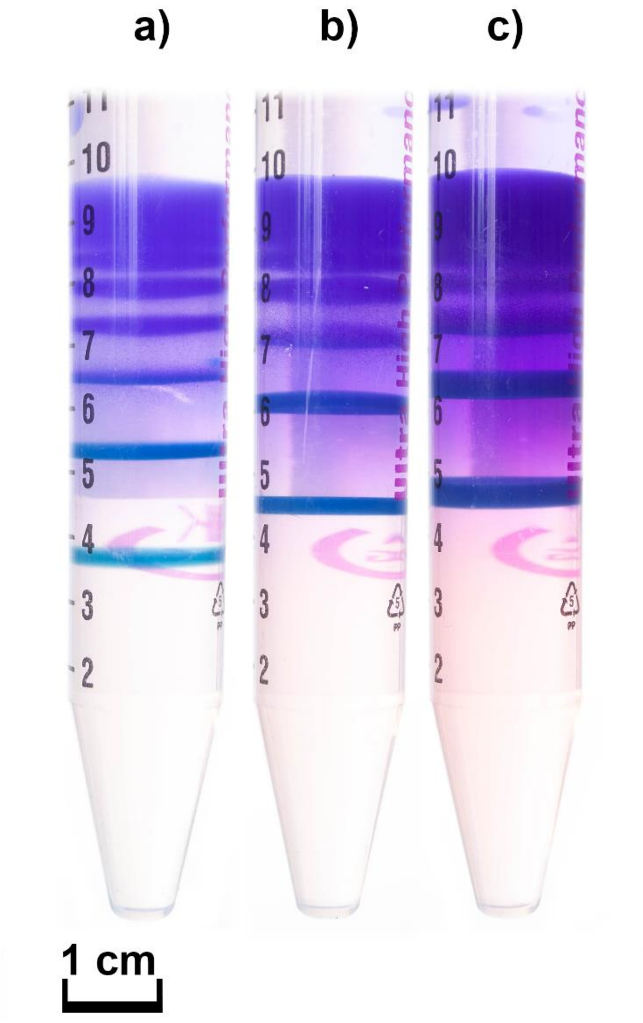

Figure S5 Periodic precipitation of ZIF-67 in the gel matrix after 1 week of the reaction and diffusion of ZIF precursors at room temperature using Falcon® tubes. The cobalt cations were homogeneously distributed in the agarose gel (0.5% m/V, DMF/H_2O volumetric ratio was 1:1). (a) $[Co^{2+}]_0$ = 5 mM, (b) $[Co^{2+}]_0$ = 10 mM, and (c) $[Co^{2+}]_0$ = 20 mM. The concentration of the outer electrolyte (2-met) was 1.0 M.

References

(1) Liu, X. D.; Tokura, S.; Haruki, M.; Nishi, N.; Sakairi, N. Surface Modification of Nonporous Glass Beads with Chitosan and Their Adsorption Property for Transition Metal Ions, *Carbohydr. Polym.*, **2002**, *49*, 103−108.

(2) Thomas, J. W. *Numerical Partial Differential Equations: Finite Difference Methods*; Springer New York, New York, 2010.
