# Supplementary material for: Formation of Precipitation Ellipsoidal Disks and Spheres in the Wake of a Planar Diffusion Front
Source: J Phys Chem Lett. 2023 Nov 13;14(46):10382–7. doi: 10.1021/acs.jpclett.3c02295 (PMC10683069; doi:10.1021/acs.jpclett.3c02295)
Supplement: Supplementary file 2 — jz3c02295_si_002.pdf [file jz3c02295_si_002.pdf]

jz-2023-02295y.R1

Name: Peer Review Information for "Formation of Precipitation Ellipsoidal Disks and Spheres in the Wake of a Planar Diffusion Front"

First Round of Reviewer Comments

Reviewer: 1

Comments to the Author

This paper reports a new type of precipitation pattern formation (ellipsoidal disks and spheres) of ZIF-67 in hydrogel column in a test tube, suggesting an important role of the surface interaction of test tube with Co-2-met complexes. The major advance reported in this paper is the discovery of an important role of the surface interaction of test tube with the intermediates of precipitates. This provides some new insights on precipitation patterning. Therefore, this paper is potentially publishable at JPCL.

Meanwhile, JPCL requires making clear what the immediate significance of the reported advance. Such immediate significance seems to be unclear in this version, and hence, the authors should emphasis it in the revised manuscript. In my opinion, if some specific properties (e.g., extremely small particle sizes favorable for use as catalyst [1]) are found (or, at least suggested) in the ZIF-67 nanoparticles consisting of the disks/spheres, they could materialize the required significance.

Additionally, the authors should make clear what the “a”, “b”, “c”, and “d” mean in the caption of Figure 2.

[1] W Xia et al, J. Mater. Chem. A, 2014, 2, 11606.

Reviewer: 2

Comments to the Author

The paper by Lagzi and coworkers reports about the formation of peculiar precipitation patterns (ellipses and spheres) during the formation of cobalt-based MOFs in a gelled medium. The manuscript is clearly written and the experimental results are supported by numerical simulations. The explanation of the

observed pattern is convincing and linear and the authors could prove their hypothesis through simple experiments.

I think the paper can be published in JPCL after a few issues will be addressed:

- Do the authors think that the type of macroscopic patterns (bands, ellipse, sphere...) can influence the properties of the crystals (size, surface area, shape, etc.)?

- P. 7 lines 13-18: the authors report the pKa value of 2-met in water to support the fact that the complex is positively charged. However, some DMF is used as the co-solvent and it is known that DMF favors the deprotonation of 2-met. Can the authors comment about that?

- p. 7 line 24: degreasing --> decreasing

Author's Response to Peer Review Comments:

István Lagzi
Associate Professor
Budapest University of Technology and Economics
Institute of Physics

H-1111, Budafoki út 8.
Budapest, Hungary
Tel: +36 1463-1341
WWW: nimbus.elte.hu/~lagzi
Email: lagzi.istvan.laszlo@ttk.bme.hu

October 11, 2023

Prof.
Senior Editor

Dear Professor ,

Thank you for sending us the very helpful comments on our manuscript entitled *„**Formation of Precipitation Ellipsoidal Disks and Spheres in the Wake of a Planar Diffusion Front**”*. In the following, we provide our point-by-point answers to the specific queries. For clarity, the *Reviewers' comments and questions are formatted in italics*.

To fully address the Reviewer's comments (Reviewer #1 comment 1 and Reviewer #2 comment 1) and improve the scientific presentation, we performed an investigation using scanning electron microscopy (SEM) to characterize the formed crystals of ZIF-67. We have involved one colleague (Dr. Gabor Schuszter, University of Szeged, Szeged, Hungary), who performed the additional SEM measurements. Now, his name is listed as a co-author of this manuscript. All original authors of the manuscript have approved his authorship. I would like to ask you for your editorial consent to add Gabor Schuszter as a co-author.

Manuscript Formatting Request - Non-scientific changes

"1) Abstract: Shorten the abstract to 150 words or fewer."

Author reply: We have shortened the abstract.

Changes: Page 2 (Word count: 150)
Pattern formation is one of the examples of self-organization. In the generation of patterns, the coupling between the mass transport of the chemical species and their chemical reaction plays an important role. Periodic precipitation (Liesegang phenomenon) is a type of pattern formation, in which layered precipitation structures form in the wake of the diffusion front. Here we show a new type of precipitation pattern formation in zeolitic imidazolate framework-67 in a solid hydrogel column in a test tube manifesting in the generation of precipitation ellipsoidal disks and spheres in the wake of the planar diffusion front of the outer electrolyte (2-methylimidazole). To increase the probability of the emergence of ellipsoidal disks and spheres, the surface of the borosilicate test tubes was chemically treated and functionalized. To support the experimental findings, we developed a reaction-diffusion model that qualitatively describes the formation of precipitation ellipsoidal disks and spheres in a test tube.

*"2) References: In both the main file and the supporting information, fix the style of all references to use JPCL formatting (check all references carefully). ***JPC Letters reference formatting requires that journal references should contain: () around numbers; author names; article title (titles entirely in title case or entirely in lower case); abbreviated journal title (italicized); year (bolded); volume (italicized); and pages (first-last). Book references should contain author names; book title (in the same pattern); publisher; city; and year. Websites must include date of access."*

"3) References: URLs are not preferred references because website content can be modified and, consequently, the reference information may lack permanence."

Author reply: The style of all references has been checked and updated.

Reviewer 1

"This paper reports a new type of precipitation pattern formation (ellipsoidal disks and spheres) of ZIF-67 in hydrogel column in a test tube, suggesting an important role of the surface interaction of test tube with Co-2-met complexes. The major advance reported in this paper is the discovery of an important role of the surface interaction of test tube with the intermediates of precipitates. This provides some new insights on precipitation patterning. Therefore, this paper is potentially publishable at JPCL."

Author reply: We thank the Reviewer for his/her constructive comments.

"Meanwhile, JPCL requires making clear what the immediate significance of the reported advance. Such immediate significance seems to be unclear in this version, and hence, the authors should emphasis it in the revised manuscript. In my opinion, if some specific properties (e.g., extremely small particle sizes favorable for use as catalyst [1]) are found (or, at least suggested) in the ZIF-67 nanoparticles consisting of the disks/spheres, they could materialize the required significance.

[1] W Xia et al, J. Mater. Chem. A, 2014, 2, 11606.*"*

Author reply: We thank for the Reviewer this comment. The main aim of the study was to investigate the formation of macroscopic patterns generated in the wake of the planar diffusion front in a precipitation reaction by applying various treatments/functionalization of the surface of the glass test tube. Based on the Reviewer's comment, we have analyzed and characterized the formed crystals by scanning electron microscopy (SEM). To fulfil the Reviewer's request, (i) we highlighted the main finding in the text, namely, the generation of precipitation disks and spheres in the wake of a planar chemical front was due to a symmetry breaking generated by the surface of the test tubes; (ii) we provided the results and discussion of SEM measurements on the size and shape of the generated crystals in parallel bands and spheres using native borosilicate glass test tubes and functionalized by chitosan. Figure 2 was extended showing the results of SEM measurements, and (iii) we cited the paper provided by the Reviewer to strengthen the scientific content of the manuscript. (iv) We also described the sample preparation and SEM measurement in the Supporting Information.

Changes:
(i) Page 11
In this study, we presented an unexpected pattern formation in a reaction-diffusion system, namely the formation of precipitation ellipsoidal disks and spheres in the wake of the planar diffusion front of the outer electrolyte (2-met). The generation of precipitation disks and spheres was due to a symmetry breaking generated by the surface of the test tubes. We hypothesized that the formed intermediate species (having a positive charge) interact with the surface of the wall of the test tube.

(ii) and (iii)
Pages 8 and 9
To get insight into the microscopic structure of the formed ZIF-8 crystals to use them in various applications (e.g., catalyst, gas storage, and drug delivery),[49] we performed scanning electron microscopy (SEM) measurements (see SI for the sample preparation and description of the measurement). Panels i-v in Figure 2 show the SEM micrographs of the formed particles in a native borosilicate test tube and test tube whose inner surface was functionalized with chitosan. In both cases, the crystals generated near the liquid-gel interface were small and amorphous (Figure 2, Panels i and iv). However, farther from the interface well-shaped crystals were formed. We compared the average size and dispersity of the particles (characterized by the standard deviation of the samples) in both cases at the same distance measured from the liquid–gel interface where the precipitation spheres were produced (~ 2cm). We found that the parallel precipitation bands consist of crystals with the size of 19.2 ± 7.5 µm (Figure 2, Panel ii), however, the precipitation spheres contain particles having slightly lower dispersity with the size of 15.9 ± 6.1 µm (Figure 2, Panel v).

In the case of a native borosilicate tube, the dispersity of the ZIF-67 crystals increased in the distance measured from the liquid–gel interface (Figure 2, Panels i-iii). This finding is in good accordance with the results obtained in other systems (formation of inorganic particles and gold nanoparticles).[50, 51]

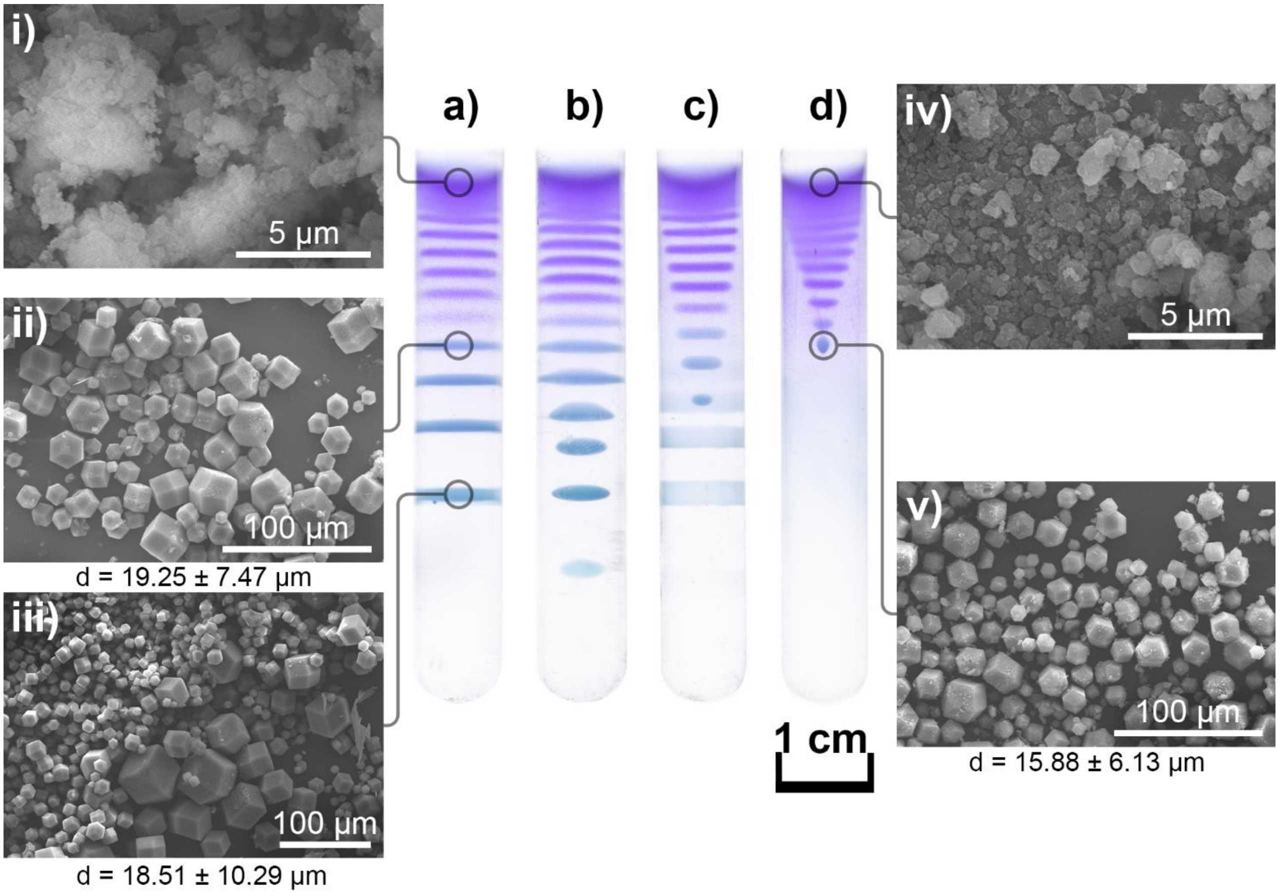

Figure 2. Periodic precipitation of ZIF-67 in the gel matrix after 1 week of the reaction and diffusion of ZIF precursors at room temperature: a) the native borosilicate test tube, b) borosilicate test tube treated with a piranha solution, c) borosilicate test tube treated with a piranha solution followed by the treatment using a sodium hydroxide solution, and d) borosilicate test tube treated with a piranha and sodium hydroxide solutions followed by the functionalization with chitosan. The cobalt cations ($[Co^{2+}]_0$ = 5 mM) were homogeneously distributed in the agarose gel (0.5% m/v, DMF/H_2O volumetric ratio was 1:1). The concentration of the outer electrolyte (2-met) was 1.0 M. Panels i-v show the SEM micrographs of the ZIF-67 crystals extracted from the domains of the agarose gel indicated in a) and d).

(49) Xia, W.; Zhu, J.; Guo, W.; An, L.; Xia, D.; Zou, R. Well-Defined Carbon Polyhedrons Prepared from Nano Metal–Organic Frameworks for Oxygen Reduction. *J. Mater. Chem. A* **2014**, *2*, 11606–11613.

(50) Walliser, R. M.; Boudoire, F.; Orosz, E.; Tóth, R.; Braun, A.; Constable, E. C.; Rácz, Z.; Lagzi, I. Growth of Nanoparticles and Microparticles by Controlled Reaction-Diffusion Processes. *Langmuir* **2015**, *31*, 1828–1834.
(51) Farkas, S.; Holló, G.; Schuszter, G.; Deák, Á.; Janovák, L.; Hornok, V.; Itatani, M.; Nabika, H.; Horváth, D.; Tóth, Á.; Lagzi, I. Reaction–Diffusion Assisted Synthesis of Gold Nanoparticles: Route from the Spherical Nano-Sized Particles to Micrometer-Sized Plates. *J. Phys. Chem. C* **2021**, *125*, 26116–26124.

(iv) SI, Pages S4

1.5 Samples preparation for scanning electron microscopy (SEM) measurement

The test tube containing the gel column was scratched with a glass cutting tool, and the tube was carefully broken. The gel was slid out and sliced up at the appropriate heights with a razor blade. The pieces of gel were put into Eppendorf tubes (2 mL) and dissolved in 1.5 mL DMF. The solution was centrifuged for 15 minutes at 10000 rpm (Hettich ROTOFIX 32A) and the supernatant was carefully removed. The remaining precipitate was suspended in 1.5 mL DMF and centrifuged again. These cleaning steps were repeated three times. After the final centrifugation step, the samples were dried at ambient conditions.

1.6 SEM measurement

The formed crystals were investigated via SEM (Hitachi S4700) by applying a secondary electron detector (Everhart–Thornley detector) and 20 kV accelerating voltage. The dry precipitate was placed onto conducting tape and gold sputtering was performed to maintain appropriate electrical conductance.

"Additionally, the authors should make clear what the "a", "b", "c", and "d" mean in the caption of Figure 2."

Author reply: We thank the Reviewer for this issue. This is a valid point; we provided the missing description in the caption.

Changes: Pages 8 and 9, Figure 2

Figure 2. Periodic precipitation of ZIF-67 in the gel matrix after 1 week of the reaction and diffusion of ZIF precursors at room temperature: a) the native borosilicate test tube, b) borosilicate test tube treated with a piranha solution, c) borosilicate test tube treated with a piranha solution followed by the treatment using a sodium hydroxide solution, and d) borosilicate test tube treated with a piranha and sodium hydroxide solutions followed by the functionalization with chitosan. The cobalt cations ($[Co^{2+}]_0$ = 5 mM) were homogeneously distributed in the agarose gel (0.5% m/v, DMF/H_2O volumetric ratio was 1:1). The concentration of the outer electrolyte (2-met) was 1.0 M. Panels i-v show the SEM micrographs of the ZIF-67 crystals extracted from the domains of the agarose gel indicated in a) and d).

Budapest University of Technology and Economics
Faculty of Natural Sciences • Institute of Physics
Fizika Tanszék
1111 Budapest, Budafoki út 8. F.épület, Hungary
Phone: +36 1463-2312, Fax: +36 1463 4180
http://dept.phy.bme.hu

Reviewer 2

"The paper by Lagzi and coworkers reports about the formation of peculiar precipitation patterns (ellipses and spheres) during the formation of cobalt-based MOFs in a gelled medium. The manuscript is clearly written and the experimental results are supported by numerical simulations. The explanation of the observed pattern is convincing and linear and the authors could prove their hypothesis through simple experiments. I think the paper can be published in JPCL after a few issues will be addressed:"

Author reply: We thank the Reviewer for his/her comments and the positive assessment of our work.

"- Do the authors think that the type of macroscopic patterns (bands, ellipse, sphere...) can influence the properties of the crystals (size, surface area, shape, etc.)?"

Author reply: We thank for the Reviewer this comment. We have analyzed and characterized the formed crystals by scanning electron microscopy (SEM). (i) we provided the results and discussion of SEM measurements on the size and shape of the generated crystals in parallel bands and spheres using native borosilicate glass test tubes and functionalized by chitosan. Figure 2 was extended showing the results of SEM measurements. We believe this investigation strengthens the scientific content of the manuscript. (ii) We also described the sample preparation and SEM measurement in the Supporting Information.

Changes:
(i)
Pages 8 and 9

To get insight into the microscopic structure of the formed ZIF-8 crystals to use them in various applications (e.g., catalyst, gas storage, and drug delivery),[49] we performed scanning electron microscopy (SEM) measurements (see SI for the sample preparation and description of the measurement). Panels i-v in Figure 2 show the SEM micrographs of the formed particles in a native borosilicate test tube and test tube whose inner surface was functionalized with chitosan. In both cases, the crystals generated near the liquid-gel interface were small and amorphous (Figure 2, Panels i and iv). However, farther from the interface well-shaped crystals were formed. We compared the average size and dispersity of the particles (characterized by the standard deviation of the samples) in both cases at the same distance measured from the liquid–gel interface where the precipitation spheres were produced (~ 2cm). We found that the parallel precipitation bands consist of crystals with the size of 19.2 ± 7.5 μm (Figure 2, Panel ii), however, the precipitation spheres contain particles having slightly lower dispersity with the size of 15.9 ± 6.1 μm (Figure 2, Panel v). In the case of a native borosilicate tube, the dispersity of the ZIF-67 crystals increased in the distance measured from the liquid–gel interface (Figure 2, Panels i-iii). This finding is in good accordance with the results obtained in other systems (formation of inorganic particles and gold nanoparticles).[50-51]

Budapest University of Technology and Economics
Faculty of Natural Sciences • Institute of Physics
Fizika Tanszék

1111 Budapest, Budafoki út 8. F.épület, Hungary
Phone: +36 1463-2312, Fax: +36 1463 4180
http://dept.phy.bme.hu

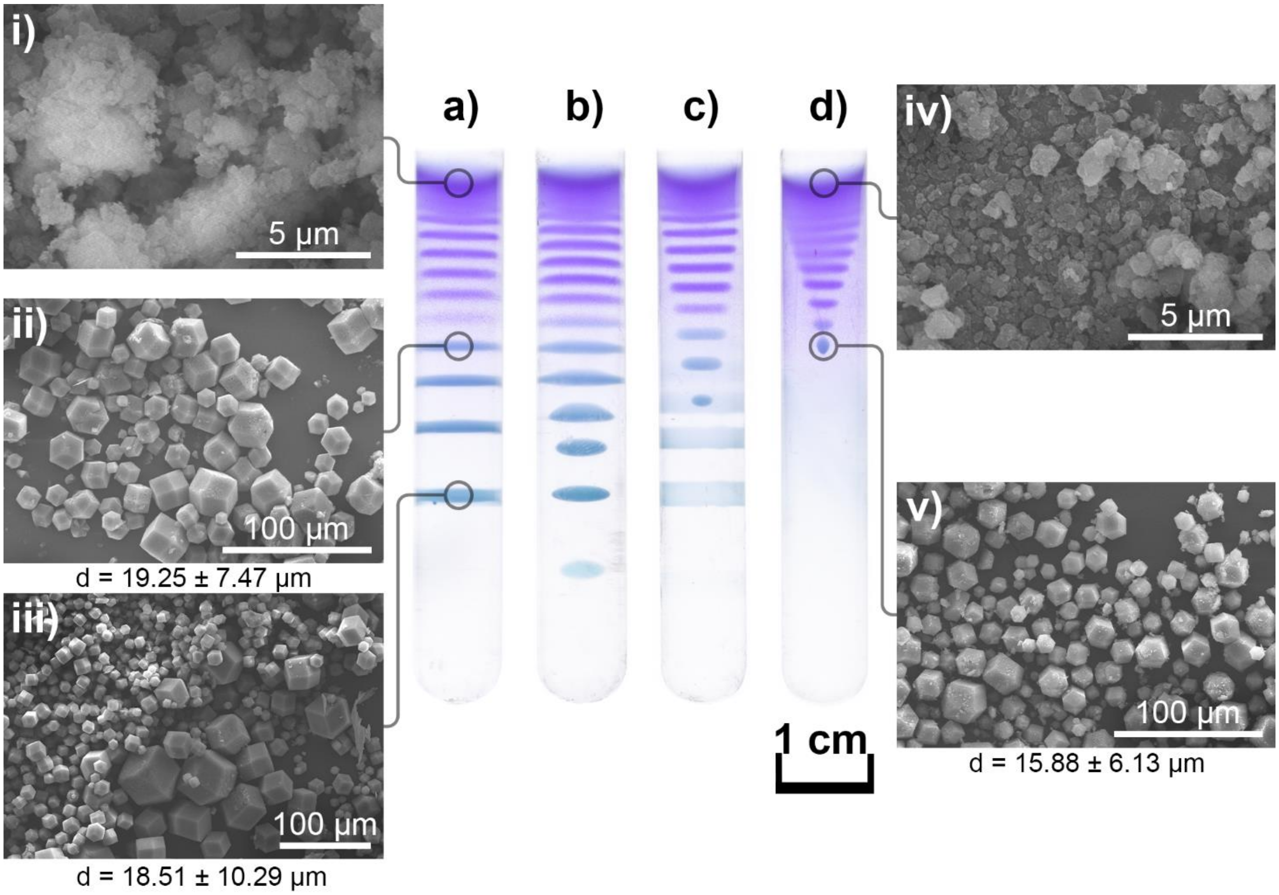

Figure 2. Periodic precipitation of ZIF-67 in the gel matrix after 1 week of the reaction and diffusion of ZIF precursors at room temperature: a) the native borosilicate test tube, b) borosilicate test tube treated with a piranha solution, c) borosilicate test tube treated with a piranha solution followed by the treatment using a sodium hydroxide solution, and d) borosilicate test tube treated with a piranha and sodium hydroxide solutions followed by the functionalization with chitosan. The cobalt cations ($[Co^{2+}]_0$ = 5 mM) were homogeneously distributed in the agarose gel (0.5% m/v, DMF/H_2O volumetric ratio was 1:1). The concentration of the outer electrolyte (2-met) was 1.0 M. Panels i-v show the SEM micrographs of the ZIF-67 crystals extracted from the domains of the agarose gel indicated in a) and d).

(49) Xia, W.; Zhu, J.; Guo, W.; An, L.; Xia, D.; Zou, R. Well-Defined Carbon Polyhedrons Prepared from Nano Metal–Organic Frameworks for Oxygen Reduction. *J. Mater. Chem. A* **2014**, *2*, 11606–11613.

(50) Walliser, R. M.; Boudoire, F.; Orosz, E.; Tóth, R.; Braun, A.; Constable, E. C.; Rácz, Z.; Lagzi, I. Growth of Nanoparticles and Microparticles by Controlled Reaction-Diffusion Processes. *Langmuir* **2015**, *31*, 1828–1834.

(51) Farkas, S.; Holló, G.; Schuszter, G.; Deák, Á.; Janovák, L.; Hornok, V.; Itatani, M.; Nabika, H.; Horváth, D.; Tóth, Á.; Lagzi, I. Reaction–Diffusion Assisted Synthesis of Gold Nanoparticles: Route

from the Spherical Nano-Sized Particles to Micrometer-Sized Plates. *J. Phys. Chem. C* **2021**, *125*, 26116–26124.

(ii) SI, Pages S4

1.5 Samples preparation for scanning electron microscopy (SEM) measurement

The test tube containing the gel column was scratched with a glass cutting tool, and the tube was carefully broken. The gel was slid out and sliced up at the appropriate heights with a razor blade. The pieces of gel were put into Eppendorf tubes (2 mL) and dissolved in 1.5 mL DMF. The solution was centrifuged for 15 minutes at 10000 rpm (Hettich ROTOFIX 32A) and the supernatant was carefully removed. The remaining precipitate was suspended in 1.5 mL DMF and centrifuged again. These cleaning steps were repeated three times. After the final centrifugation step, the samples were dried at ambient conditions.

1.6 SEM measurement

The formed crystals were investigated via SEM (Hitachi S4700) by applying a secondary electron detector (Everhart–Thornley detector) and 20 kV accelerating voltage. The dry precipitate was placed onto conducting tape and gold sputtering was performed to maintain appropriate electrical conductance.

"- P. 7 lines 13-18: the authors report the pKa value of 2-met in water to support the fact that the complex is positively charged. However, some DMF is used as the co-solvent and it is known that DMF favors the deprotonation of 2-met. Can the authors comment about that?"

Author reply: This is a valid point. DMF can indirectly influence the pKa of a solute using a mixture of water and DMF as a solvent because DMF can solvate and stabilize ions or polar molecules differently compared to water. DMF can weaken or strengthen the acidity or basicity of a compound due to the stabilization or destabilization of the charged or uncharged forms of the solute differently from water. We hypothesize that the presence of DMF decreases the pKa of 2-met, but in the generated solvent environment most of the 2-met molecules are still protonated. To highlight it, we added a short discussion in the text.

In addition to this, pure DMF can dissolve the agarose hydrogel (in other words, no gelation can be observed), however, the used 1:1 ratio (DMF: water) provided a solid agarose gel. The effect of the DMF content on the macroscopic pattern in ZIF-8 and ZIF-67 systems was investigated in our previous paper: Farkas *et al*., Periodic Precipitation of Zeolitic Imidazolate Frameworks in a Gelled Medium. *J. Phys. Chem. C* **2022**, *126*, 9580–9586.

Changes: Page 7

This complex has a positive charge ($[Co(2\text{-met})_4]^{2+}$) because in water the pKa of 2-met is ~14 meaning that practically no 2-met molecules are deprotonated, therefore, they are electrically neutral.[44] It is known that DMF (and other organic solvents) can weaken or strengthen the acidity or basicity of a chemical species due to the stabilization or destabilization of the charged or uncharged

Budapest University of Technology and Economics
Faculty of Natural Sciences • Institute of Physics
Fizika Tanszék

1111 Budapest, Budafoki út 8. F.épület, Hungary
Phone: +36 1463-2312, Fax: +36 1463 4180
http://dept.phy.bme.hu

forms of the solute differently from water. However, we hypothesize that the presence of DMF decreases the pKa of 2-met, but in the generated solvent environment most of the 2-met molecules are still neutral.[45]

(45) Rossini, E.; Bochevarov, A. D.; Knapp, E. W. Empirical Conversion of pKa Values between Different Solvents and Interpretation of the Parameters: Application to Water, Acetonitrile, Dimethyl Sulfoxide, and Methanol. *ACS Omega* **2018**, *3*, 1653–1662.

"- p. 7 line 24: degreasing --> decreasing"

Author reply: We corrected this misprint in the text.

Changes: Page 7
The positively charged intermediate can adsorb on the negatively charged glass surface, decreasing its concentration near the wall of the test tube.

This completes our reply. We again thank the Referees for their comments/suggestions and critical reading of our work. We hope that the manuscript is now in an acceptable form.

Sincerely yours,

István Lagzi

Budapest University of Technology and Economics
Faculty of Natural Sciences ● Institute of Physics
Fizika Tanszék

1111 Budapest, Budafoki út 8. F.épület, Hungary
Phone: +36 1463-2312, Fax: +36 1463 4180
http://dept.phy.bme.hu
